# The correlation and predictive value of Hb, RDW and their association for short-term and long-term mortality in patients with acute aortic dissection

**DOI:** 10.3389/fcvm.2024.1444498

**Published:** 2025-01-06

**Authors:** Peng Hou, Lin Xia, Fangran Xin, Boxuan Sun, Guoxin Zhang, Liming Yu, Huishan Wang

**Affiliations:** ^1^ Department of Cardiovascular Surgery, General Hospital of Northern Theater Command, Shenyang, Liaoning, China; ^2^ 65052 troops, The Chinese People’s Liberation Army, Taonan, Jilin, China

**Keywords:** aortic dissection, hemoglobin, red blood cell distribution width, mortality risk, prognostic biomarkers

## Abstract

**Background:**

This study examines the relationship between Hb, RDW and their association and both short-term and long-term mortality in patients with acute aortic dissection (AAD), aiming to establish combined effect between Hb and RDW as a potential prognostic biomarker for AAD outcomes.

**Methods:**

We extracted clinical data from the Medical Information Mart for Intensive Care (MIMIC) databases for this analysis. Using adjusted Cox regression and Kaplan-Meier survival curve analyses, we assessed the relationship between Hb, RDW and their association at admission and mortality at multiple post-discharge intervals (30 days, 90 days, 1 year, and 5 years) among patients with AAD. Additionally, subgroup analyses and receiver operating characteristic (ROC) curve analyses were conducted to evaluate the predictive accuracy of Hb, RDW and their association for mortality in this patient population.

**Results:**

High RDW combined with low Hb (RDW ≥ 13.60 and Hb < 7.9) significantly predicted increased mortality at 30 days, 90 days, 1 year, and 5 years post-diagnosis, with hazard ratios (HRs) as follows: 4.33 (95% CI: 1.82–10.33, *P* < 0.001), 4.48 (95% CI: 2.06–9.77, *P* < 0.001), 3.38 (95% CI: 1.70–6.70, *P* < 0.001), and 3.07 (95% CI: 1.66–5.66, *P* < 0.001), respectively.

**Conclusion:**

Hb and RDW are both abnormal (Hb with low level, RDW with high level) is positively correlated with 30 days, 90 days, 1 year, and 5 years mortality risk in patients with AAD. This suggests that combined effect between Hb and RDW is a significant predictor of short-term to long-term mortality risk in this patient population, highlighting its potential utility as a prognostic marker in clinical settings.

## Introduction

Acute aortic dissection (AAD) represents a critical cardiovascular condition characterized by high mortality rates (1). It is estimated that the risk of death is 1%–2% per hour, and the mortality rate of non-surgical patients can reach up to 60% (2, 3). The early identification of patients at high risk and the implementation of effective risk stratification management are essential for enhancing the prognosis of AAD and alleviating the overall disease burden (1–3). In this context, the search for simple yet accurate biomarkers is pivotal for effective risk stratification.

Anemia is frequently encountered in the intensive care unit (ICU) and is strongly linked to increased mortality risks among these patients (4, 5). For those with AAD, aneurysmal blood flow into the false lumen may lead to a reduction in circulating blood volume, often detectable as anemia via routine blood tests (2). Studies have demonstrated that diminished hemoglobin (Hb) levels correlate with adverse short-term outcomes in type A AAD (6). Similarly, in patients with type B AAD, lower Hb levels have been associated with higher risks of all-cause mortality and major cardiovascular events following endovascular aortic repair (TEVAR) (7, 8). Moreover, an increase in red blood cell distribution width (RDW), which reflects the variability in the size of circulating red blood cells, is commonly seen in patients with anemia (9). RDW is influenced by various factors including oxidative stress, endothelial dysfunction, and inflammation, and is linked to diverse cardiovascular outcomes (10). Elevated oxidative stress levels in AAD patients can directly damage red blood cells, reducing their lifespan and increasing cellular heterogeneity and RDW (11). Previous research indicates that high RDW levels are linked to increased mortality following TEVAR, and this association persists even after adjustments for Hb levels (12). The combined metric of Hb and RDW, known as HRR, integrates prognostic data from both markers, offering a more robust prediction than either parameter alone (13). Although the prognostic value of HRR has been explored in other diseases such as coronary heart disease (14, 15) and ischemic stroke (16), and even cancer (17), its relevance in predicting outcomes in AAD patients remains understudied. Theoretically speaking, when Hb and RDW are both abnormal (Hb with low level, RDW with high level), the risk of death seems to be the highest. Simultaneously focusing on these two indicators is valuable for risk stratification and prognosis prediction. So further exploration is needed to determine whether Hb, RDW and their association can predict the outcome of AAD.

## Methods

### Database source

This study employed a retrospective design, utilizing data from the publicly accessible Medical Information Mart for Intensive Care (MIMIC) database. The MIMIC III dataset comprises adult patient records from intensive care unit admissions spanning 2001–2012, while MIMIC IV extends this coverage from 2008 to 2019 (18, 19). The MIMIC database was established in 2003 with funding from the National Institutes of Health (NIH). It was collaboratively developed by a diverse team of professionals including emergency and intensive care physicians, computer scientists, and researchers from the Beth Israel Deaconess Medical Center, the Massachusetts Institute of Technology (MIT), Oxford University, and the Massachusetts General Hospital (MGH). The MIMIC database encompasses a broad range of data categories, such as demographic information, insurance details, ethnicity, and clinical interventions, including detailed clinical observations, laboratory tests, physiological scores, administered medications, surgical procedures, and survival outcomes (20).

### Inclusion and exclusion criteria

#### Inclusion criteria

This study included individuals aged 18 years or older who were diagnosed with AAD. Eligibility was contingent upon the availability of complete data for Hb and RDW.

#### Exclusion criteria

Patients were excluded if they had incomplete survival data or if their stay in the ICU was less than 24 h.

### Data stratification and definitions

Acute aortic dissection patients: ICD9 starting with 4410, ICD10 starting with I710. HRR was defined as Hb divided by RDW. The outcome variables assessed were mortality at 30 days, 90 days, 1 year, and 5 years post-diagnosis. And type A aortic dissection refers to a category of AAD that includes patients who require surgical interventions such as cardiovascular surgery, valve repair, or operations on the heart's vessels. In analyzing this condition, we identified covariates from the data presented in Table 1; variables with a *P*-value of less than 0.05 were selected as confounding factors for this study.

**Table 1:** Distribution of data.

Variables		Death			
	Total (*n* = 700)	No (*n* = 487)	Yes (*n* = 213)	Statistics	*P*
Age, Year, Mean ± SD	66.37 ± 14.64	64.23 ± 14.37	71.25 ± 14.11	*t* = −5.98	**<**.**001**
Gender, *n* (%)				*χ^2^* = 1.300	0.254
Female	280 (40.00)	188 (38.60)	92 (43.19)		
Male	420 (60.00)	299 (61.40)	121 (56.81)		
Insurance, *n* (%)				*χ^2^* = 11.076	**<**.**001**
Medicare	364 (52.00)	233 (47.84)	131 (61.50)		
Others	336 (48.00)	254 (52.16)	82 (38.50)		
Congestive heart failure, *n* (%)				*χ^2^* = 11.904	**<**.**001**
No	564 (80.57)	409 (83.98)	155 (72.77)		
Yes	136 (19.43)	78 (16.02)	58 (27.23)		
Diabetes, *n* (%)				*χ^2^* = 0.289	0.591
No	615 (87.86)	430 (88.30)	185 (86.85)		
Yes	85 (12.14)	57 (11.70)	28 (13.15)		
Hypertension, *n* (%)				*χ^2^* = 1.582	0.208
No	291 (41.57)	210 (43.12)	81 (38.03)		
Yes	409 (58.43)	277 (56.88)	132 (61.97)		
Marfan syndrome, *n* (%)				–	1.000
No	690 (98.57)	480 (98.56)	210 (98.59)		
Yes	10 (1.43)	7 (1.44)	3 (1.41)		
Renal disease, *n* (%)				*χ^2^* = 10.138	**0**.**001**
No	586 (83.71)	422 (86.65)	164 (77.00)		
Yes	114 (16.29)	65 (13.35)	49 (23.00)		
Coronary artery disease, *n* (%)				*χ^2^* = 9.652	**0**.**002**
No	565 (80.71)	408 (83.78)	157 (73.71)		
Yes	135 (19.29)	79 (16.22)	56 (26.29)		
Stroke, *n* (%)				*χ^2^* = 7.445	**0**.**006**
No	652 (93.14)	462 (94.87)	190 (89.20)		
Yes	48 (6.86)	25 (5.13)	23 (10.80)		
Stanford type, *n* (%)				*χ^2^* = 4.919	**0**.**027**
A	55 (7.86)	31 (6.37)	24 (11.27)		
Other	645 (92.14)	456 (93.63)	189 (88.73)		
Vasopressor, *n* (%)				*χ^2^* = 4.605	**0**.**032**
No	378 (54.00)	276 (56.67)	102 (47.89)		
Yes	322 (46.00)	211 (43.33)	111 (52.11)		
RRT, *n* (%)				*χ^2^* = 18.497	**<**.**001**
No	620 (88.57)	448 (91.99)	172 (80.75)		
Yes	80 (11.43)	39 (8.01)	41 (19.25)		
Mechanical ventilation, *n* (%)				*χ^2^* = 4.334	**0**.**037**
No	159 (22.71)	100 (20.53)	59 (27.70)		
Yes	541 (77.29)	387 (79.47)	154 (72.30)		
SAPSII, M (Q_1_, Q_3_)	36.00 (29.00, 45.00)	35.00 (28.00, 43.00)	39.00 (32.00, 47.00)	*Z* = 4.322	**<**.**001**
GCS, M (Q_1_, Q_3_)	15.00 (13.00, 15.00)	15.00 (13.00, 15.00)	14.00 (11.00, 15.00)	*Z* = −1.943	0.052
qSOFA, M (Q_1_, Q_3_)	2.00 (2.00, 2.00)	2.00 (2.00, 2.00)	2.00 (2.00, 3.00)	*Z* = 1.471	0.141
Heart rate, BPM, Mean ± SD	80.43 ± 15.72	80.06 ± 14.77	81.28 ± 17.73	*t* = −0.88	0.381
Systolic, mmHg, Mean ± SD	123.18 ± 23.79	122.15 ± 22.78	125.53 ± 25.85	*t* = −1.65	0.100
Diastolic, mmHg, Mean ± SD	64.58 ± 15.26	63.82 ± 14.64	66.32 ± 16.50	*t* = −1.91	0.056
Respiratory rate, Mean ± SD	16.95 ± 4.83	16.53 ± 4.49	17.93 ± 5.40	*t* = −3.32	**<**.**001**
WBC, K/ul, M (Q1, Q3)	10.00 (7.60, 12.90)	10.10 (7.90, 12.70)	9.80 (7.10, 13.30)	*Z* = −0.415	0.678
Platelet, K/ul, M (Q1, Q3)	161.50 (121.00, 214.00)	163.00 (120.00, 214.00)	161.00 (122.00, 218.00)	*Z* = 0.098	0.922
Hemoglobin, g/dl, Mean ± SD	10.70 ± 2.12	10.81 ± 2.11	10.45 ± 2.11	*t* = 2.10	**0**.**036**
RDW, %, Mean ± SD	14.38 ± 1.58	14.14 ± 1.41	14.92 ± 1.81	*t* = −5.55	**<**.**001**
Hematocrit, %, Mean ± SD	31.91 ± 6.26	32.18 ± 6.26	31.30 ± 6.24	*t* = 1.72	0.085
Creatinine, mg/dl, M (Q_1_, Q_3_)	1.00 (0.80, 1.30)	1.00 (0.80, 1.30)	1.10 (0.80, 1.50)	*Z* = 2.422	**0**.**015**
Glucose, mg/dl, M (Q_1_, Q_3_)	131.00 (109.00, 159.00)	130.00 (108.00, 158.00)	131.00 (110.00, 161.00)	*Z* = 0.588	0.556
Sodium, mEq/L, Mean ± SD	137.88 ± 3.53	137.83 ± 3.33	138.00 ± 3.95	*t* = −0.54	0.593
Chloride, mEq/L, Mean ± SD	105.29 ± 4.72	105.14 ± 4.46	105.65 ± 5.26	*t* = −1.25	0.212
Potassium, mg/dl, Mean ± SD	4.29 ± 0.86	4.29 ± 0.87	4.28 ± 0.84	*t* = 0.03	0.973
Calcium, mg/dl, M (Q_1_, Q_3_)	7.40 (1.11, 8.60)	2.00 (1.11, 8.50)	7.80 (1.14, 8.60)	*Z* = 1.228	0.219
Magnesium, mg/dl, Mean ± SD	2.18 ± 0.55	2.19 ± 0.54	2.15 ± 0.59	*t* = 0.81	0.419
Cancer, *n* (%)				*χ^2^* = 2.285	0.131
No	472 (67.43)	337 (69.20)	135 (63.38)		
Yes	228 (32.57)	150 (30.80)	78 (36.62)		
Hematological diseases, *n* (%)				*χ^2^* = 1.095	0.295
No	390 (55.71)	265 (54.41)	125 (58.69)		
Yes	310 (44.29)	222 (45.59)	88 (41.31)		
HRR, Mean ± SD	0.76 ± 0.18	0.78 ± 0.18	0.71 ± 0.17	*t* = 4.27	**<**.**001**

Abbreviations: SD, standard deviation; SAPSII, simplified acute physiology score Ⅱ; GCS, Glasgow coma scale; qSOFA, quick sepsis related organ failure assessment; WBC, white blood cell; RDW, red blood cell distribution width; RRT, renal replacement therapy; HRR, hemoglobin to red cell distribution width ratio.

Bold values represent statistical significance.

### Statistical analysis

Multiple imputation method is used for missing variables. Maximally Selected Rank Statistics was used for the classification cut-off points of Hb and RDW. The classification method of HRR is based on the Third quartile.

#### Descriptive analysis

Continuous variables normally distributed are expressed as mean ± standard deviation (SD) and analyzed using the two-sample *t*-test. Non-normally distributed continuous variables are presented as median (Q1, Q3) and evaluated using the two-sample rank sum test. Categorical variables are reported as counts and percentages (*n*, %) and analyzed using the chi-square test or Fisher's exact test when appropriate.

#### Univariate analysis

We conducted univariate analyses using the *t*-test, rank sum test, chi-square test, or Fisher's exact test as outlined in Table 1. Additionally, univariate Cox regression and logistic regression analyses were performed, with results visualized in Table 2 and the area under the curve (AUC) plots, respectively.

**Table 2:** HRs (95% CIs) for mortality across groups of Hb, RDW and their association.

Variables	30-day mortality		90-day mortality		1-year mortality		5-year mortality	
	HR (95% CI)	*P*	HR (95% CI)	*P*	HR (95% CI)	*P*	HR (95% CI)	*P*
Model 1								
HRR								
<0.67	Ref		Ref		Ref		Ref	
0.67–0.83	0.97 (0.60–1.56)	0.892	1.06 (0.70–1.60)	0.772	0.94 (0.66–1.36)	0.752	0.92 (0.66–1.27)	0.602
>0.83	0.42 (0.23–0.76)	**0**.**005**	0.42 (0.25–0.71)	**0**.**001**	0.44 (0.28–0.68)	**<0**.**001**	0.51 (0.35–0.74)	**<0**.**001**
HB								
<7.9	Ref		Ref		Ref		Ref	
≥7.9	0.48 (0.28–0.84)	**0**.**011**	0.51 (0.31–0.84)	**0**.**008**	0.66 (0.41–1.06)	0.085	0.73 (0.47–1.12)	0.144
RDW								
<13.60	Ref		Ref		Ref		Ref	
≥13.60	3.09 (1.64–5.83)	**<0**.**001**	3.70 (2.07–6.59)	**<0**.**001**	3.37 (2.08–5.46)	**<0**.**001**	2.91 (1.96–4.33)	**<0**.**001**
RDW and HB								
<13.60 and ≥7.9	Ref		Ref		Ref		Ref	
<13.60 and <7.9	1.73 (0.22–13.52)	0.601	1.46 (0.19–11.19)	0.718	0.97 (0.13–7.26)	0.976	2.01 (0.61–6.65)	0.252
≥13.60 and ≥7.9	2.92 (1.49–5.73)	**0**.**002**	3.50 (1.91–6.42)	**<0**.**001**	3.24 (1.96–5.33)	**<0**.**001**	3.03 (1.99–4.60)	**<0**.**001**
≥13.60 and <7.9	5.37 (2.38–12.09)	**<0**.**001**	6.00 (2.89–12.45)	**<0**.**001**	4.37 (2.29–8.32)	**<0**.**001**	3.45 (1.94–6.13)	**<0**.**001**
*P* for trend	**<0.001**		**<0.001**		**<0.001**		**<0.001**	
Model 2								
HRR								
<0.67	Ref		Ref		Ref		Ref	
0.67–0.83	0.99 (0.60–1.65)	0.974	1.08 (0.70–1.68)	0.722	0.93 (0.63–1.36)	0.703	0.88 (0.62–1.23)	0.442
>0.83	0.67 (0.34–1.29)	0.228	0.63 (0.35–1.11)	0.110	0.58 (0.36–0.93)	**0**.**024**	0.60 (0.40–0.90)	**0**.**013**
HB								
<7.9	Ref		Ref		Ref		Ref	
≥7.9	0.55 (0.30–1.00)	0.050	0.59 (0.34–1.01)	0.053	0.73 (0.44–1.21)	0.222	0.72 (0.45–1.14)	0.158
RDW								
<13.60	Ref		Ref		Ref		Ref	
≥13.60	2.53 (1.31–4.91)	**0**.**006**	2.84 (1.56–5.16)	**<0**.**001**	2.62 (1.60–4.30)	**<0**.**001**	2.37 (1.58–3.56)	**<0**.**001**
RDW and HB								
<13.60 and ≥7.9	Ref		Ref		Ref		Ref	
<13.60 and <7.9	1.20 (0.15–9.85)	0.868	1.11 (0.14–8.76)	0.924	0.84 (0.11–6.38)	0.862	2.05 (0.60–6.94)	0.251
≥13.60 and ≥7.9	2.36 (1.18–4.74)	**0**.**016**	2.68 (1.43–5.00)	**0**.**002**	2.51 (1.50–4.18)	**<0**.**001**	2.45 (1.60–3.77)	**<0**.**001**
≥13.60 and <7.9	4.33 (1.82–10.33)	**<0**.**001**	4.48 (2.06–9.77)	**<0**.**001**	3.38 (1.70–6.70)	**<0**.**001**	3.07 (1.66–5.66)	**<0**.**001**
*P* for trend	**0.001**		**<0.001**		**<0**.**001**		**<0**.**001**	

Abbreviations: HR, hazard ratio; CI, confidence interval; HRR, hemoglobin to red cell distribution width ratio; Hb, hemoglobin; RDW, red blood cell distribution width.

Model1: adjust: None.

Model2: adjust: Age, Insurance, Congestive heart failure, Renal disease, Coronary artery disease, Stroke, Stanford type, Vasopressor, RRT, Mechanical ventilation, SAPSII, GCS, Respiratory rate, Creatinine, Cancer, Hematological diseases.

Bold values represent statistical significance.

#### Multivariate analysis

Covariates were adjusted for in the multivariate Cox regression analyses to confirm the stability of the correlation between Hb, RDW and their association form and mortality. This approach was also applied to subgroup analyses to explore the association further. Key evaluation metrics included hazard ratios (HRs), confidence intervals, AUC values, and Delong test.

#### Statistical confidence and software

The significance level was set at alpha = 0.05. Data cleaning was performed using SAS 9.4 (SAS Institute Inc., Cary, NC, USA), and statistical analyses were conducted using R software, version 4.3.0.

## Results

### Characteristics of the subjects

**Figure 1:**
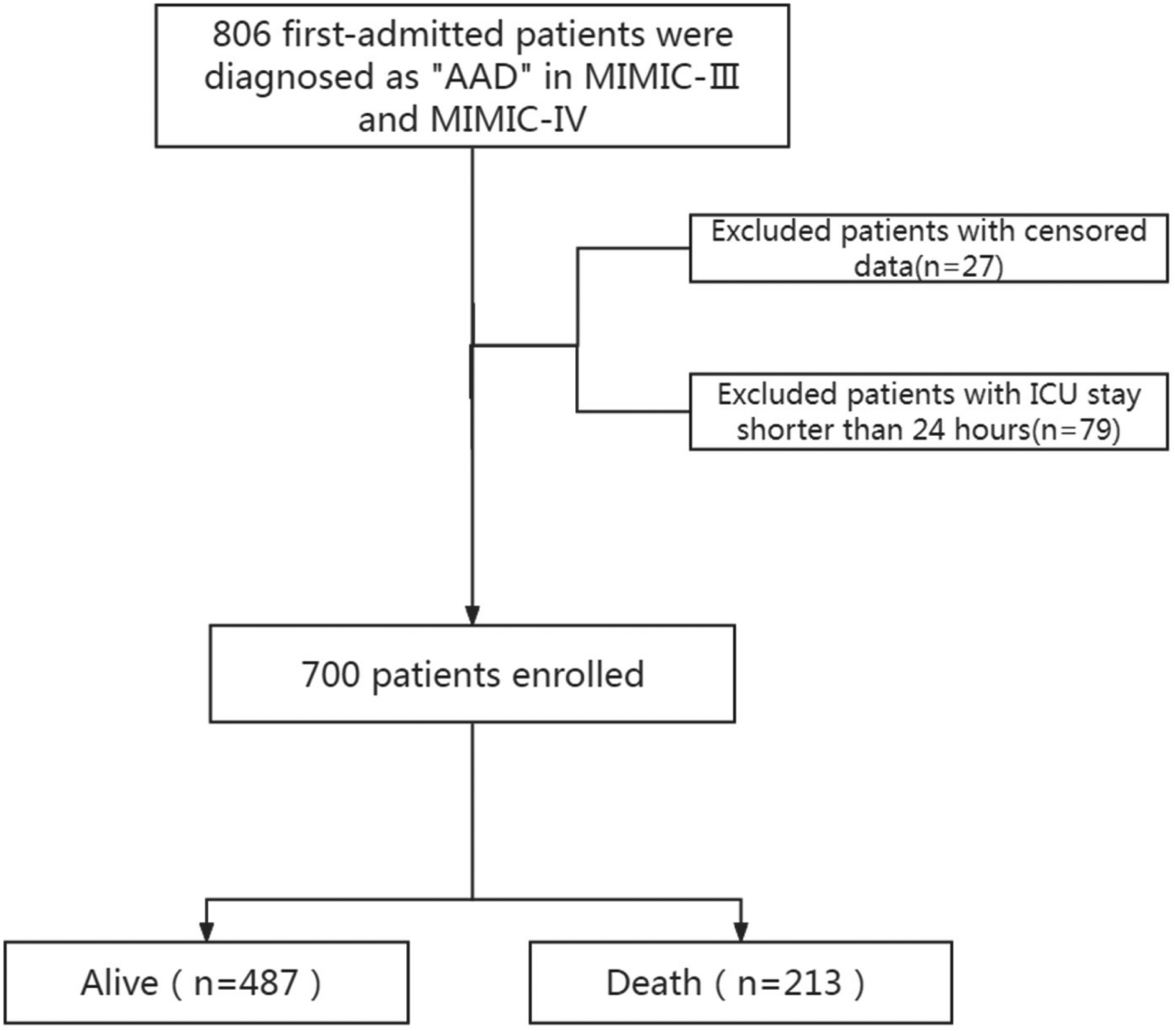
Screening process diagram. AAD, acute aortic dissection; MIMIC, medical information mart for intensive care; ICU, intensive care unit.

The study included 700 patients diagnosed with AAD, as detailed in the patient selection flowchart (Figure 1). There are 26 Hb missing samples and 27 RDW missing samples. The baseline characteristics of these patients are summarized in Table 1. Patients were categorized into two groups based on survival outcomes: those who survived (alive group) and those who did not (death group).

Comparative analysis revealed that patients in the death group were generally older and more likely to suffer from a range of comorbidities including congestive heart failure, renal disease, and coronary artery disease. Additionally, these patients exhibited a higher prevalence of stroke, RRT (renal replacement therapy), mechanical ventilation, and had elevated respiratory rates and creatinine levels. Higher incidences of cancer, hematological diseases, and RDW were also observed in the death group. Conversely, Hb levels and HRR were notably lower in patients who did not survive.

### High HRR is directly related to medium to long-term mortality of AAD

**Figure 2:**
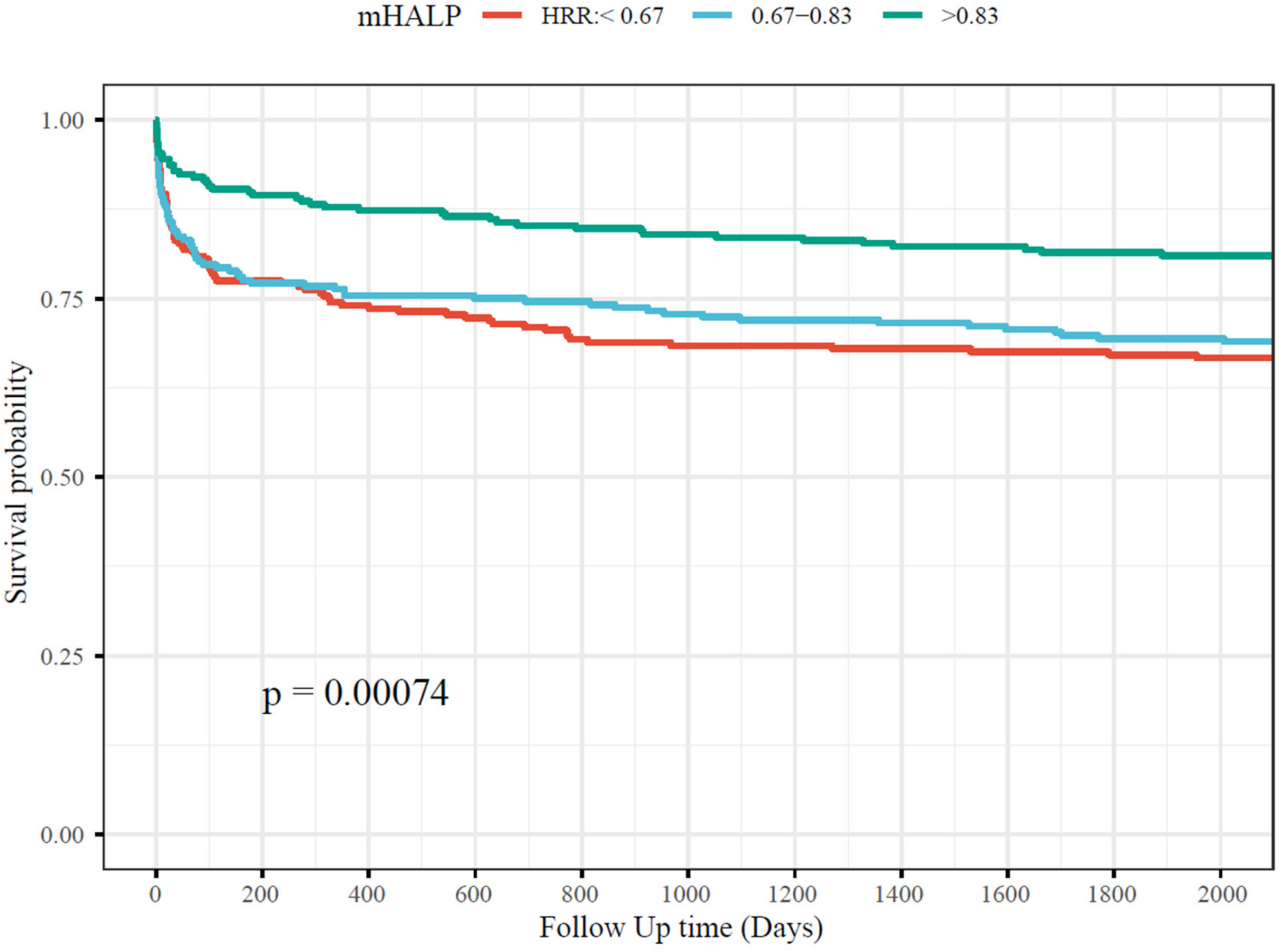
The association between HRR and short-term and long-term mortality. HRR, hemoglobin to red cell distribution width ratio.

Over the five-year follow-up period, 213 (30.43%) of the 700 patients with AAD succumbed to the condition. Survival analyses depicted in the Kaplan–Meier curve (Figure 2) illustrate that patients in the high HRR group experienced significantly better survival rates compared to those in the mid and low HRR groups.

To further delineate the relationship between HRR and mortality, multivariate Cox regression analyses were performed, as detailed in Table 2. Initially, the analyses were conducted based on the results from covariate selection. In Model 1, without any adjustment, high HRR (greater than 0.67) significantly predicted reduced mortality at 30 days, 90 days, 1 year, and 5 years post-diagnosis, with hazard ratios (HRs) and 95% confidence intervals (CIs) as follows: 0.42 (0.23–0.76), 0.42 (0.25–0.71), 0.44 (0.28–0.68), and 0.51 (0.35–0.74), respectively.

In Model 2, after adjusting for age, insurance status, congestive heart failure, renal disease, coronary artery disease, stroke, Stanford type, use of vasopressors, RRT, mechanical ventilation, SAPS II score, GCS score, respiratory rate, creatinine levels, cancer, and hematological diseases, high HRR still remained a significant predictor of reduced 1-year and 5-year mortality, with HRs of 0.58 (0.36–0.93) and 0.60 (0.40–0.90), respectively. Furthermore, subgroup analyses that explored the relationship between admission HRR levels and mortality at intervals of 30 days, 90 days, 1 year, and 5 years post-diagnosis are detailed in Supplementary Table 1.

### The combined effect between Hb and RDW is an effective predictor of short to long-term mortality of AAD

In order to further explore the relationship of the combined effect between Hb and RDW with mortality, multivariate Cox regression analyses were also performed, as detailed in Table 2. In Model 2, high RDW combined with low Hb (RDW ≥ 13.60 and Hb < 7.9) significantly predicted increased mortality at 30 days, 90 days, 1 year, and 5 years post-diagnosis, with HRs and 95% CIs as follows: 4.33 (1.82–10.33), 4.48 (2.06–9.77), 3.38 (1.70–6.70), and 3.07 (1.66–5.66), respectively.

**Figure 3:**
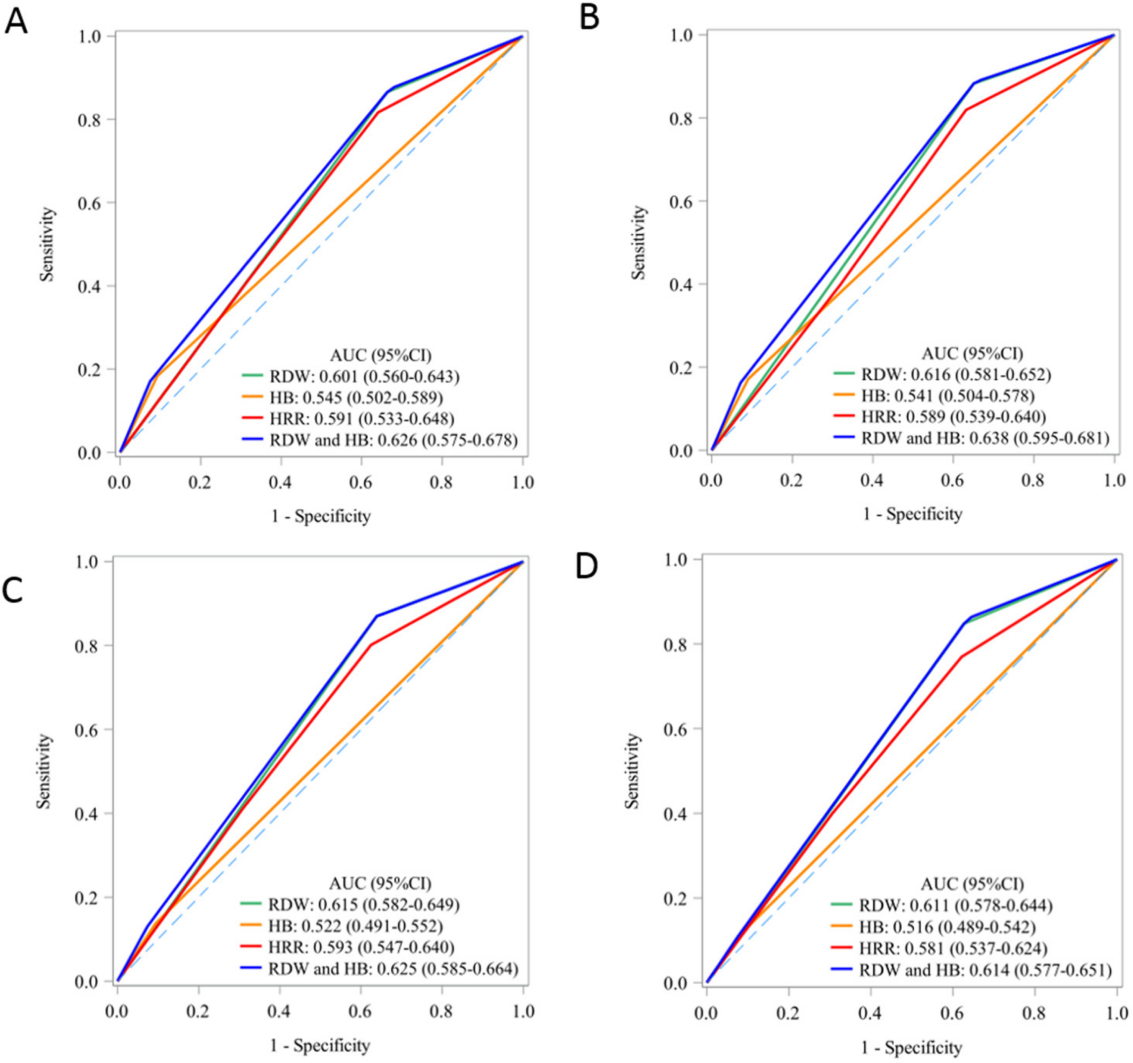
The predictive value of Hb, RDW and their association for short-term and long-term mortality: AUC curve. AUC, area under curve; HB, hemoglobin; RDW, red blood cell distribution width; HRR, hemoglobin to red cell distribution width ratio. **(A)** 30-day mortality; **(B)** 90-day mortality; **(C)** 1-year mortality; **(D)** 5-year mortality.

The area under the curve (AUC) for the combined Hb and RDW level in predicting mortality across various time points (30 days, 90 days, 1 year, and 5 years) ranged between 0.61 and 0.63. The AUC of the combined effect of Hb and RDW at each time point was better than that of Hb and RDW as well as HRR (Figure 3).

**Figure 4:**
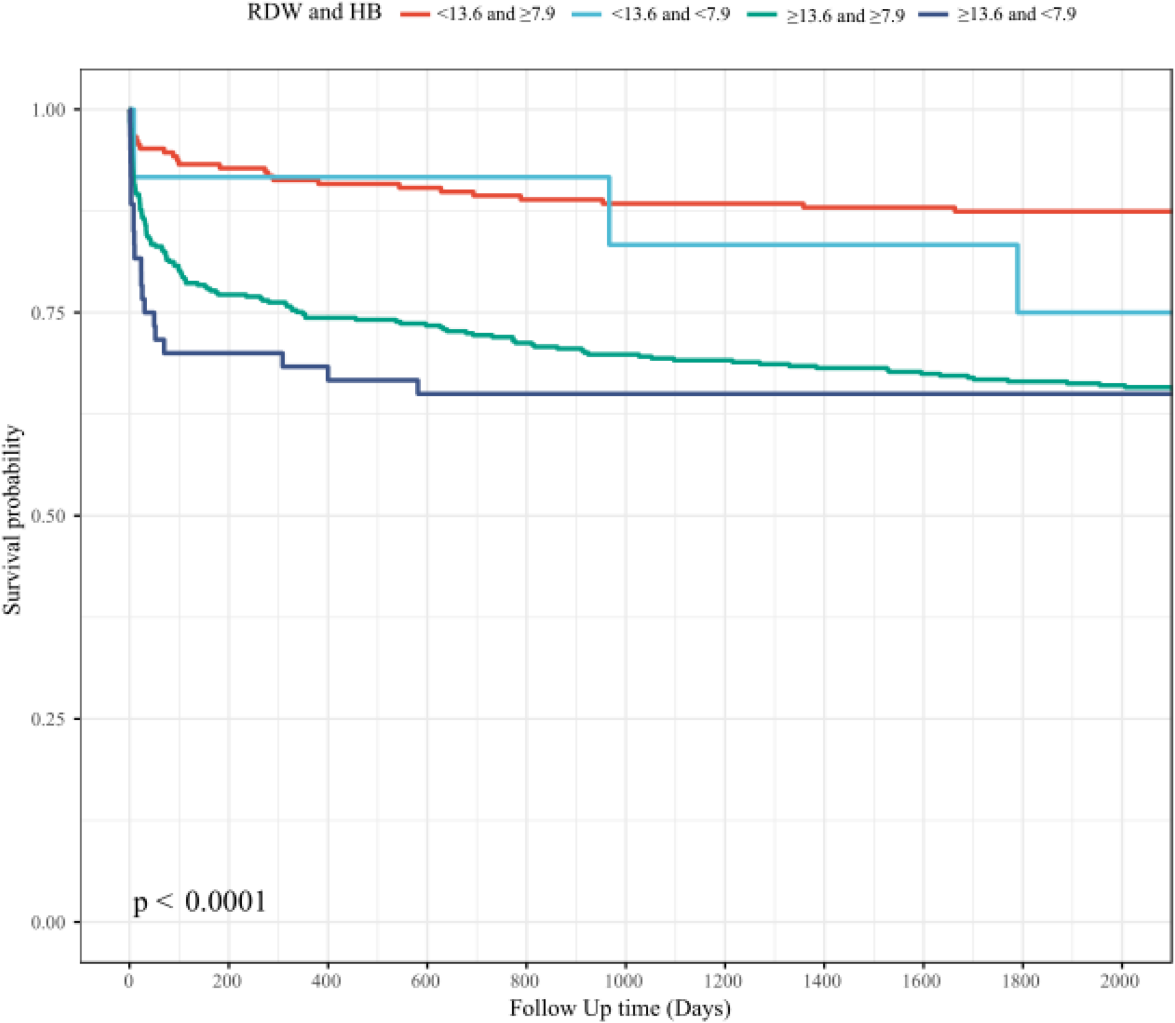
The association between combined effect of Hb and RDW and short-term and long-term mortality: HB, hemoglobin; RDW, red blood cell distribution width.

Survival analyses (Figure 4) illustrate that patients in the high RDW combined with low Hb (RDW ≥ 13.60 and Hb < 7.9) group experienced significantly lowest survival rates compared to those in the other combined effect of Hb and RDW groups.

Additionally, we have tested the combined effect to improve the existing score, and added a Delong test of interaction term + existing score vs. existing score to demonstrate the significance of our research, as detailed in Table 3.

**Table 3:** Comparison of AUC between CE and SAPSII, qSOFA.

	Variables	AUC (95% CI)	Statistic	*P*
30-day mortality	SAPSII	0.666 (0.602–0.730)	*χ^2^* = 4.448	Ref
	CE + SAPSII	0.702 (0.641–0.763)		**0.035**
	qSOFA	0.605 (0.548–0.662)	*χ^2^* = 12.0165	Ref
	CE + qSOFA	0.681 (0.623–0.739)		**0.0005**
90-day mortality	SAPSII	0.644 (0.589–0.699)	*χ^2^* = 8.4692	Ref
	CE + SAPSII	0.695 (0.644–0.747)		**0.0036**
	qSOFA	0.587 (0.536–0.638)	*χ^2^* = 20.2818	Ref
	CE + qSOFA	0.684 (0.634–0.734)		**<0.0001**
1-year mortality	SAPSII	0.629 (0.579–0.679)	*χ^2^* = 10.8533	Ref
	CE + SAPSII	0.682 (0.635–0.729)		**0.001**
	qSOFA	0.564 (0.517–0.612)	*χ^2^* = 20.9712	Ref
	CE + qSOFA	0.661 (0.615–0.707)		**<0.0001**
5-year mortality	SAPSII	0.597 (0.551–0.644)	*χ^2^* = 16.5145	Ref
	CE + SAPSII	0.652 (0.608–0.696)		**<0.0001**
	qSOFA	0.527 (0.483–0.571)	*χ^2^* = 26.8673	Ref
	CE + qSOFA	0.631 (0.588–0.674)		**<0.0001**

Abbreviations: AUC, area under curve; CE, combined effect; CI, confidence interval; SAPSII, simplified acute physiology score Ⅱ; qSOFA, quick sepsis related organ failure assessment.

Bold values represent statistical significance.

## Discussion

AAD is a severe cardiovascular condition marked by sudden onset, rapid progression, and a high mortality rate (2). Currently, emergency surgical intervention, particularly Sun's surgery (21)—which includes total arch replacement and stent graft insertion (elephant trunk procedure)—is widely advocated and has gained consensus among cardiovascular specialists (2). Although advancements in medical technology have significantly reduced mortality rates from AAD surgery, these rates and the incidence of complications remain comparatively high against other cardiovascular interventions such as those for coronary heart disease and congenital heart disease (1–3). Total arch replacement surgery involves extended extracorporeal circulation times and requires low-temperature circulatory arrest, presenting substantial risks and physical trauma to patients, thereby classifying it as high-risk within cardiac surgical procedures (2). It is essential to provide objective, reliable, and readily accessible clinical data for early postoperative risk assessment and to initiate targeted monitoring and intervention strategies.

In clinical practice, the widely used perioperative mortality and adverse prognosis risk scoring systems, such as EuroSCORE II (22) and APACHE II (23), are not specifically tailored for AAD patients. EuroSCORE II is designed for all cardiac surgeries, whereas APACHE II is broad, covering all ICU patients without special consideration for cardiac surgery cases, particularly those involving AAD total arch replacement. Both systems suffer from complexities in their scoring processes, requiring substantial clinical data input, which diminishes their practicality. The Leipzig Halifax scoring system (24), introduced in 2016, offers a simpler approach and is specifically designed for AAD. However, its predictive capabilities still require further validation due to its recent development and the small, primarily Western sample size on which it is based.

Our study findings indicate that combined effect between Hb and RDW shows significant predictive value for short-term to long-term mortality risk in AAD patients. High RDW combined with low Hb (RDW ≥ 13.60 and Hb < 7.9) significantly predicted increased mortality at 30 days, 90 days, 1 year, and 5 years post-diagnosis, with HRs as follows: 4.33 (95% CI: 1.82–10.33, *P* < 0.001), 4.48 (95% CI: 2.06–9.77, *P* < 0.001), 3.38 (95% CI: 1.70–6.70, *P* < 0.001), and 3.07 (95% CI: 1.66–5.66, *P* < 0.001), respectively, suggesting that Hb and RDW are both abnormal (Hb with low level, RDW with high level) is positively correlated with mortality risk.

Besides, our study also finds that HRR as divided into three categories based on quartiles shows significant predictive value for medium to long-term mortality risk in AAD patients. With an HRR less than 0.67 as a reference, the HRs for one-year and five-year mortality were 0.58 (95% CI: 0.36–0.93, *P* = 0.024) and 0.60 (95% CI: 0.40–0.90, *P* = 0.013) respectively, suggesting that higher HRR levels are inversely correlated with mortality risk. These results align with existing literature on the prognostic value of HRR in other cardiovascular diseases, such as coronary heart disease and ischemic stroke. Studies like those by Qu et al. (15) on coronary heart disease in the elderly, and Chen et al. (13) on acute heart failure patients, have similarly highlighted HRR as a robust predictor of adverse outcomes.

It is worth mentioning that AUC of the combined effect of Hb and RDW at each time point was better than that of Hb and RDW as well as HRR, while AUC of HRR is not better than that of RDW (Hb was the worst). This leaves HRR still a doubt in AAD patients' prognostic assessment, and large-scale, prospective studies are needed in the future.

Promoting joint application of Hb and RDW in AAD patients is both feasible and reliable for several reasons. Firstly, such as HRR, as a composite marker, provides a more comprehensive assessment of disease severity than individual indicators (13). It primarily serves as a marker of anemia dysfunction—where severe anemia corresponds to lower Hb levels, higher RDW, and subsequently, lower HRR. Moreover, HRR data is straightforward to collect and calculate from routine follow-ups, providing a more objective and reliable metric compared to EuroSCORE II and APACHE II. Additionally, HRR offers real-time, dynamic data post-surgery, unlike other measurements that may suffer from delays and dependency on specific equipment or personnel. Given its objective and quantitative nature, HRR could potentially become the primary evaluation indicator in future large-scale vessel research, offering uniformity and comparability across studies.

### Limitation

This study has several limitations:

Data Source and External Validation: The patient data utilized in this study was sourced from public databases. While this provided a substantial dataset and supported conclusions that combined effect between Hb and RDW shows significant predictive value for short-term to long-term mortality risk in AAD patients, the lack of external validation currently limits these findings. Future efforts will involve using data from our own patient cohorts for external validation to confirm whether combined effect between Hb and RDW can effectively predict both long-term and short-term mortality in AAD as well. Additional multicenter, prospective studies with larger sample sizes are needed to further validate its predictive capacity for mortality in AAD. Given its ease of acquisition, such validation could also extend to medical big data platforms, including multicenter databases like the International Registry of Acute Aortic Dissection (IRAD) (25), potentially enhancing the explanatory power of the results.

Scope of Conclusions: In this study, there were only 55 patients with type A ADD, too little to investigate outcomes based on the type of dissection (A-B), and failed to differentiate the results between treated and not treated patients, therefore also failed to identify a possible predictive value of the combined effect between Hb and RDW to the surgery/treated. Further research involving larger models and datasets is necessary to identify the predictive role of combined effect between Hb and RDW on prognosis in different AAD subgroups.

## Conclusion

In conclusion, our findings demonstrate that Hb and RDW are both abnormal (Hb with low level, RDW with high level) is positively correlated with 30 days, 90 days, 1 year, and 5 years mortality risk in patients with AAD. This suggests that combined effect between Hb and RDW is a significant predictor of short-term to long-term mortality risk in this patient population, highlighting its potential utility as a prognostic marker in clinical settings.

## Data Availability

The datasets presented in this study can be found in online repositories. The names of the repository/repositories and accession number(s) can be found in the article/Supplementary Material.
