# Supplementary material for: The correlation and predictive value of Hb, RDW and their association for short-term and long-term mortality in patients with acute aortic dissection
Source: Front Cardiovasc Med. 2025 Jan 6;11:1444498. doi: 10.3389/fcvm.2024.1444498 (PMC11743433; doi:10.3389/fcvm.2024.1444498)
Supplement: Supplementary file 1 [file Table1.pdf]

SUPPLEMENTARY TABLE 1 Subgroup analysis of the association with admission HRR and mortality

Model 2

Variables		30-day mortality		90-day mortality		1-year mortality		5-year mortality	
		HR (95%CI)	*P*	HR (95%CI)	*P*	HR (95%CI)	*P*	HR (95%CI)	*P*
Age < 65	HRR								
	<0.67	Ref		Ref		Ref		Ref	
	0.67-0.83	0.60 (0.18-1.97)	0.395	0.71 (0.29-1.76)	0.462	0.85 (0.39-1.86)	0.684	0.68 (0.33-1.39)	0.291
	>0.83	0.50 (0.14-1.74)	0.274	0.41 (0.14-1.22)	0.110	0.50 (0.21-1.21)	0.124	0.56 (0.26-1.20)	0.137
Age >= 65	HRR								
	<0.67	Ref		Ref		Ref		Ref	
	0.67-0.83	1.24 (0.69-2.26)	0.471	1.26 (0.75-2.11)	0.390	0.96 (0.61-1.50)	0.844	0.90 (0.61-1.33)	0.595
	>0.83	0.61 (0.27-1.39)	0.236	0.60 (0.29-1.23)	0.164	0.50 (0.28-0.93)	**0.027**	0.52 (0.32-0.87)	**0.012**
Gender: Female	HRR								
	<0.67	Ref		Ref		Ref		Ref	
	0.67-0.83	2.19 (1.00-4.81)	0.050	2.05 (1.00-4.20)	0.051	1.62 (0.88-2.98)	0.118	1.19 (0.69-2.06)	0.539
	>0.83	1.21 (0.41-3.62)	0.729	0.77 (0.27-2.24)	0.636	0.69 (0.30-1.60)	0.387	0.58 (0.28-1.22)	0.150
Gender: Male	HRR								
	<0.67	Ref		Ref		Ref		Ref	
	0.67-0.83	0.47 (0.21-1.05)	0.065	0.62 (0.33-1.15)	0.131	0.52 (0.30-0.90)	**0.020**	0.60 (0.38-0.97)	**0.037**
	>0.83	0.45 (0.19-1.08)	0.073	0.48 (0.24-0.99)	**0.047**	0.43 (0.24-0.80)	**0.007**	0.51 (0.30-0.84)	**0.009**
Insurance: Medicare	HRR								
	<0.67	Ref		Ref		Ref		Ref	
	0.67-0.83	1.98 (1.01-3.89)	**0.047**	1.94 (1.06-3.57)	**0.032**	1.48 (0.87-2.49)	0.146	1.16 (0.74-1.83)	0.516
	>0.83	0.84 (0.32-2.21)	0.727	0.71 (0.29-1.73)	0.450	0.67 (0.32-1.43)	0.303	0.72 (0.40-1.30)	0.275
Insurance: Others	HRR								
	<0.67	Ref		Ref		Ref		Ref	
	0.67-0.83	0.35 (0.13-0.98)	**0.045**	0.62 (0.29-1.34)	0.226	0.58 (0.31-1.11)	0.100	0.62 (0.35-1.09)	0.099
	>0.83	0.46 (0.17-1.24)	0.125	0.50 (0.22-1.13)	0.097	0.45 (0.23-0.88)	**0.020**	0.48 (0.26-0.87)	**0.016**
Hypertension: No	HRR								
	<0.67	Ref		Ref		Ref		Ref	
	0.67-0.83	1.42 (0.63-3.21)	0.400	1.45 (0.74-2.84)	0.279	1.28 (0.71-2.29)	0.406	1.14 (0.66-1.97)	0.644

	>0.83	0.32 (0.08-1.21)	0.093	0.24 (0.08-0.79)	**0.018**	0.39 (0.17-0.90)	**0.028**	0.50 (0.25-1.02)	0.056
Hypertension: Yes	HRR								
	<0.67	Ref		Ref		Ref		Ref	
	0.67-0.83	0.90 (0.46-1.78)	0.766	0.92 (0.50-1.69)	0.780	0.76 (0.45-1.28)	0.299	0.76 (0.48-1.18)	0.220
	>0.83	1.15 (0.52-2.56)	0.735	1.18 (0.58-2.38)	0.647	0.81 (0.44-1.49)	0.498	0.70 (0.42-1.16)	0.171
Mechanical ventilation: No	HRR								
	<0.67	Ref		Ref		Ref		Ref	
	0.67-0.83	0.66 (0.09-4.85)	0.683	2.66 (0.46-15.36)	0.273	1.13 (0.42-3.03)	0.805	0.76 (0.36-1.59)	0.462
	>0.83	1.17 (0.13-10.79)	0.888	2.59 (0.34-19.88)	0.359	0.71 (0.20-2.57)	0.605	0.43 (0.17-1.06)	0.066
Mechanical ventilation: Yes	HRR								
	<0.67	Ref		Ref		Ref		Ref	
	0.67-0.83	0.90 (0.52-1.59)	0.726	0.97 (0.60-1.57)	0.901	0.93 (0.60-1.43)	0.743	0.90 (0.60-1.34)	0.606
	>0.83	0.43 (0.19-0.99)	**0.047**	0.45 (0.23-0.90)	**0.023**	0.53 (0.30-0.93)	**0.028**	0.63 (0.38-1.02)	0.062
Vasopressor: No	HRR								
	<0.67	Ref		Ref		Ref		Ref	
	0.67-0.83	1.93 (0.64-5.84)	0.243	2.98 (1.03-8.62)	**0.044**	1.68 (0.82-3.43)	0.153	1.06 (0.61-1.84)	0.843
	>0.83	1.16 (0.34-4.04)	0.810	1.44 (0.45-4.65)	0.540	0.96 (0.43-2.11)	0.911	0.70 (0.38-1.29)	0.255
Vasopressor: Yes	HRR								
	<0.67	Ref		Ref		Ref		Ref	
	0.67-0.83	0.65 (0.33-1.28)	0.216	0.71 (0.40-1.27)	0.247	0.74 (0.44-1.25)	0.26	0.81 (0.50-1.32)	0.401
	>0.83	0.45 (0.18-1.11)	0.082	0.46 (0.21-1.02)	0.057	0.48 (0.23-0.97)	**0.040**	0.57 (0.30-1.09)	0.088
Cancer: No	HRR								
	<0.67	Ref		Ref		Ref		Ref	
	0.67-0.83	1.19 (0.63-2.25)	0.594	1.33 (0.75-2.35)	0.332	1.13 (0.69-1.84)	0.633	1.10 (0.70-1.73)	0.669
	>0.83	0.46 (0.19-1.13)	0.092	0.52 (0.24-1.10)	0.089	0.51 (0.28-0.94)	**0.032**	0.61 (0.37-1.03)	0.062
Cancer: Yes	HRR								
	<0.67	Ref		Ref		Ref		Ref	
	0.67-0.83	0.70 (0.25-1.94)	0.494	0.88 (0.38-2.01)	0.760	0.79 (0.38-1.64)	0.532	0.68 (0.37-1.23)	0.201
	>0.83	1.05 (0.34-3.19)	0.938	0.82 (0.31-2.18)	0.689	0.75 (0.32-1.77)	0.509	0.61 (0.30-1.23)	0.168
Hematological diseases: No	HRR								
	<0.67	Ref		Ref		Ref		Ref	
	0.67-0.83	1.25 (0.55-2.84)	0.586	1.65 (0.84-3.27)	0.148	1.68 (0.92-3.08)	0.090	1.57 (0.92-2.66)	0.096
	>0.83	1.24 (0.50-3.09)	0.642	1.28 (0.58-2.82)	0.536	1.23 (0.62-2.44)	0.553	1.17 (0.66-2.09)	0.591

Hematological diseases: Yes	HRR								
	<0.67	Ref		Ref		Ref		Ref	
	0.67-0.83	0.75 (0.36-1.56)	0.441	0.83 (0.43-1.60)	0.577	0.59 (0.33-1.05)	0.074	0.56 (0.33-0.95)	**0.030**
	>0.83	-	-	0.08 (0.01-0.63)	**0.016**	0.19 (0.06-0.54)	**0.002**	0.22 (0.09-0.54)	**<.001**

Abbreviations: *HR*, hazard ratio; *CI*, confidence interval; *HRR*, hemoglobin to red cell distribution width ratio.

Model2: adjust: Age, Insurance, Congestive heart failure, Renal disease, Coronary artery disease, Stroke, Stanford type, Vasopressor, RRT, Mechanical ventilation, SAPSII, GCS, Respiratory rate, Creatinine, Cancer, Hematological diseases.
